# Effects of remifentanil and remifentanil-alfentanil administration on emergence agitation after brief ophthalmic surgery in children

**DOI:** 10.1186/s12871-016-0213-2

**Published:** 2016-08-02

**Authors:** Yi Hwa Choi, Kyung Mi Kim, Soo Kyung Lee, Yi Seul Kim, Seon Ju Kim, Woon Suk Hwang, Jin Huan Chung

**Affiliations:** 1 Department of Anesthesiology and Pain Medicine, Hallym University Sacred Heart Hospital, Hallym University College of Medicine, 22 Gwanpyeong-ro, 170 beon-gil, Dongan-gu, Anyang 431-796 Republic of Korea; 2 Department of Anesthesiology and Pain Medicine, College of Medicine, Kangwon National University, Chuncheon, 200-701 Republic of Korea; 3 Department of Anesthesiology and Pain Medicine, Korea University Anam Hospital, 73 Inchon-ro Seongbuk-gu, Seoul, 02841 Republic of Korea

**Keywords:** Alfentanil, Anesthesia recovery period, Psychomotor agitation, Remifentanil, Sevoflurane

## Abstract

**Background:**

Sevoflurane is commonly usedin pediatric anesthesia due to its non-irritating airway properties, and rapid induction and emergence. However, it is associated with emergence agitation (EA) in children. EA may cause injury to the child or damage to the surgical site and is a cause of stress to both caregivers and families. The efficacy of remifentanil and additional alfentanil on EA in the pediatric patients underwent ophthalmic surgery with sevofluraneanesthesiawas not well evaluated to date. This study was designed to compare the effects of remifentanil and remifentanil plus alfentanil on EA in children undergoing ophthalmic surgery with sevofluraneanesthesia.

**Methods:**

Children (aged 3–9 years) undergoing ophthalmic surgery undersevoflurane anesthesia were randomly assigned to group S (sevoflurane alone), group R (sevofluraneandremifentanil infusion, 0.1 μg/kg/min), or group RA (sevoflurane withremifentanil infusion and intravenous injection of alfentanil 5 μg/kg 10 min before the end of surgery). Mean arterial pressure (MAP), heart rate (HR), and sevoflurane concentration were checked every 15 min after induction of anesthesia. The incidence of EA, time to extubation from discontinuation of sevoflurane inhalation, and time to discharge from the postanesthesia care unit was assessed.

**Results:**

The incidence of EA was significantly lower in groups R (32 %, 11/34; *P* = 0.01) and RA (31 %, 11/35; *P* = 0.008) than group S (64 %, 21/33). The time to extubation was prolonged in group RA (11.2 ± 2.3 min; *P* = 0.004 and *P* = 0.016) compared with groups S (9.2 ± 2.3 min) andR (9.5 ± 2.4 min). MAP and HR were similar in all three groups, apart from a reduction in HR at 45 min in groups R and RA. However, the sevoflurane concentration was lower in groups R and RA than group S (*P* < 0.001).

**Conclusions:**

The administration of remifentanil to children undergoing ophthalmic surgery undersevoflurane anesthesia reduced the incidence of EA without clinically significant hemodynamic changes. However, the addition of alfentanil(5 μg/kg)10 min before the end of surgery provided no additional benefit compared withremifentanil alone.

**Trial registration:**

Clinical trial number: NCT02486926, June.29.2015.

## Background

Emergence agitation (EA) or emergence delirium defined as “a combative, excited, and disoriented behavior that requires transient restraint during emergence from anesthesia”. It may cause delayed recovery and discharge from the post anesthesia care unit (PACU), injury to the patient and surgical site damage, makes monitoring difficult, and causes stress inboth caregivers and families [1–3].

Sevoflurane is commonly usedin pediatric anesthesia due to its non-irritating airway properties and the rapidity ofinduction and emergence from anesthesia. However, sevoflurane is associated with a relatively high incidence of EA, ranging from 10 to 80 %, in children [4, 5]. Ophthalmology procedures and surgery involving the tonsils, thyroid, and middle ear have been reported to have high incidences of EA [6]. Variouspharmacological agents, such as clonidine [5, 7], dexmedetomidine [8, 9], fentanyl [8, 10–12], propofol [12], and ketamine [13, 14], have been used to prevent and treat EA in children. However, their reported efficacies are various and there were no gold-standard regimen for decreasing the EA in pediatric patients.

Remifentanil is a potent opioid that is used widely in‘balanced’ anesthesia because of its rapid onset, very short context-sensitive half-time, and lack of accumulation. However, the effects of remifentanilon EA arestill controversial [15–18]. In addition, there were still some concerns about opioid-induced hyperalgesia in postoperative period [19, 20]. The clinical usefulness of another short-acting opioid, alfentanil, which known to decrease the incidence of EA during emergence from isoflurane anesthesia without additional postoperative side effects in adult patients [21], was not well evaluated in pediatrics.

In this regards, we sought to evaluate the main hypothesis that whether continuous remifentanil infusion decrease the incidence of EAand recovery characteristics in pediatric patients underwent ophthalmic surgery with sevoflurane anesthesia. In addition, we also testify whether the addition of alfentanilto continuous remifentanil infusion provides additional clinical benefit over remifentanil alone which leads optimization of remifentanil usage.

## Methods

This randomized, double-blind study enrolled 108 children who were classified as American Society of Anesthesiologists physical status I or II (i.e. normal/healthy or with mild systemic disease; http://www.asahq.org), aged 3–9 years, who were scheduled to undergo strabismus surgery or epiblepharon repair surgery under general anesthesia at the Department of Ophthalmology, Hallym University Sacred Heart Hospital, College of Medicine, Hallym University, Anyang, Republic of Korea.

Patients were excluded for any of the following reasons: developmental delay, neurological or psychological disease, history of sleep apnea, or history of general anesthesia.

This study was approved by the Institutional Review Board of Hallym University Sacred Heart Hospital (reference numbers: IORG0004993, IRB00005964). Written informed consent was obtained from the parents or legal guardians.

No premedication was administered. While in the operating room, all patients were monitored by standard limb lead electrocardiography, pulse oximetry, non-invasive blood pressure measurements, end-tidal anaesthetic gas concentration, and capnography(CARESCAPE Monitor B650; GE Healthcare, Helsinki, Finland). Anesthesia was induced with 5 mg/kg of thiopental sodium intravenous (i.v.). After loss of consciousness, children were ventilated with 3–3.5 vol. % of sevoflurane in oxygen via a face mask and fully relaxed with 0.6 mg/kg of rocuronium bromide i.v.. They were intubated with an endotracheal tube of appropriate size for age. A half dose of thiopental sodium was administered before transferring children to the operating room in cases of children with a separation score of 3 or 4 (Parental Separation Anxiety Scale (PSAS);1 = excellent; separation easily, 2 = good; not clinging, whimpers, calms with reassurance, 3 = fair; not clinging, will not calm or quiet, 4 = poor; crying, clinging to parent) [18]. Anesthesia was maintained with 1.5–3 % sevoflurane and air in oxygen (F_I_O_2_ 0.5). Mean arterial pressure (MAP), heart rate (HR), and end-tidal sevoflurane concentration (Et-sevo) were recorded at baseline and 15, 30, and 45 min after induction of anesthesia.

Study drugs were prepared by an anesthesiologist, who did not participate in data collection, in 3-ml and 20-ml syringes: normal saline with or without alfentanil in a total volume of 2 mlin a 3-ml syringe and normal saline 20 ml with or without 50 μg/ml of remifentanil in a 20-ml syringe. Patients were randomly allocated into one of three groups (S, R, RA) by a sealed-envelope method. Patients in group S received continuous infusion of normal saline from a 20-ml syringe (infusion rate comparable to remifentanil0.1 μg/kg/min)from induction of anesthesia to the end of surgery and 2 ml of normal saline10 min before the end of surgery. Patients in group R received continuous infusion of remifentanil(0.1 μg/kg/min) from induction to the end of surgery and 2 ml of normal saline 10 min before the end of surgery. Patients in group RA received continuous infusion of remifentanil(0.1 μg/kg/min) and 5 μg/kg of alfentanil 10 min before the end of surgery.

The concentration of sevoflurane was adjusted to maintain blood pressure and heart rate within a 20 % deviation from baseline values. Mechanical ventilation was adjusted to maintain a partial pressure of end-tidal carbon dioxide of 35–40 mmHg throughout the procedure. At the end of surgery, sevoflurane and the study drug prepared in 20-ml synringe (with or without remifentanil) were discontinued. All children received eye ointment in both eyes without eye patches. Antagonism of muscle relaxation was achieved with i.v.administration of 0.05 mg/kg neostigmine and 0.008 mg/kg glycopyrrolate. The endotracheal tube was removed when the patient displayed adequate spontaneous ventilation, motor activity, and facial grimacing. The time to extubation(ET) was defined as the time from the end of sevoflurane administration to removal of the endotracheal tube. On arrival to the post-anesthetic care unit (PACU), one of the patient’s parents stayed with their child until discharge. Patients were discharged from the PACU when the postanesthetic Aldrete recovery score was ≥ 9, and the duration of PACU stay was recorded.

Demographic data andthe duration of anesthesia were recorded for each patient. MAP, HR, and end-tidal sevoflurane concentration were recorded before induction of anesthesia and at 15, 30, and 45 min after induction of anesthesia.

The primary outcome of this study was the incidence of EA. The degree of agitation was graded on the four-point scale ofWatcha et al. [22] (1 = calm, 2 = crying, but can be consoled, 3 = crying, cannot be consoled, and 4 = agitated and thrashing around) from immediately after extubation and the highest score during emergence was used for the evaluation. For statistical purposes, grades 3 or 4 were considered EA. Alfentanil(5 μg/kg) was given for severe agitation (grade 4) lasting longer than 5 min. Secondary outcomes were the time to extubation and duration of PACU stay. All data were recordedby another anesthesiologist who did not know which syringe contained remifentaniland which containedalfentanil.

In a preliminary study conducted in 15 patients who received sevoflurane alone, 11 patients who underwent pediatric ophthalmic surgery showedEA. A sample-size calculation was made using a power analysis (α = 0.05, β = 0.8) to detect a 50 % reduction of the incidenceinEA (from 73 to 37 %) and was found to require 32 patients per group. Assuming a potential dropout rate of 10 %, the final sample size was set at 36 patients per group. The data are expressed as mean ± standard deviation or as numbers of patients. All statistical analyses were performed using the SPSS for Windows software package(SPSS Inc, IL, USA). A one-way analysis of variance (ANOVA) was employed in the intergroup comparisons of age, height, weight, duration of anesthesia, extubation time, and PACU time. Sex, numbers of patients with separation score (≥3), incidence of EA, and use of rescue drug were compared using a chi-square test. MAP, HR, and End-tidal Sevoflurane concentration were analyzed by repeated measured ANOVA and intergroup differences at the same time points were compared using one-way ANOVA. And all significant results were further analyzed with the scheff’s post hoc test. A *p*-value < 0.05 was considered to indicate statistical significance.

## Results

**Fig. 1:**
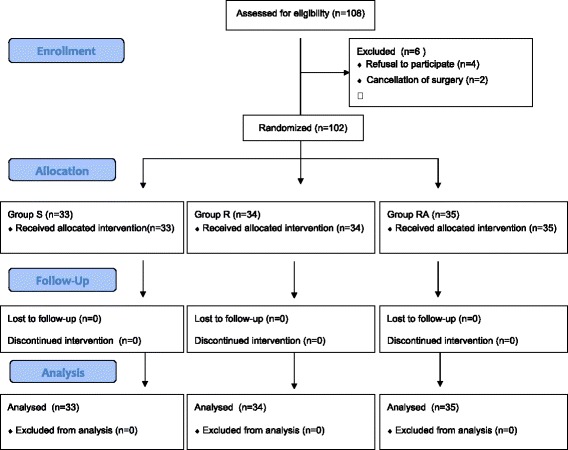
CONSORT diagram showing the flow of participants in present study

In total of 102 patients were enrolled. All patients completed the study and analyzed (Fig. 1). Demographic data, separation score, and duration of anesthesia were similar among the three groups (Table 1).

**Table 1:** Demographic characteristics, separation score, and duration of anesthesia

	Group S (*n* = 33)	Group R (*n* = 34)	Group RA (*n* = 35)	*P* value (overall)
Age (year)	6.1 ± 2.3	6.2 ± 2.0	5.8 ± 1.9	0.772
Height (cm)	117.6 ± 15.9	119.4 ± 12.0	115.5 ± 13.1	0.500
Weight (kg)	24.0 ± 10.1	24.7 ± 7.9	22.7 ± 6.1	0.576
Sex (male/female)	16/17	17/17	16/19	0.937
Separation score (≥3)	11 (33 %)	9 (26 %)	10 (29 %)	0.820
Duration of anesthesia (min)	75.3 ± 18.1	79.8 ± 17.8	78.1 ± 10.6	0.504

Data presented as mean ± SD or number of patients

Group S, patients received sevoflurane; Group R, patients received sevoflurane and continuous infusion of remifentanil; Group RA, patients received sevoflurane, continuous infusion of remifentanil, and alfentanil given 10 min before the end of surgery

Separation score before transferring children to an operating room, 1 = excellent; separation easily, 2 = good; not clinging, whimpers, calms with reassurance, 3 = fair; not clinging, will not calm or quiet, 4 = poor; crying, clinging to parent

There was no significant difference in MAP (*P* > 0.05). Reduction of the heart rate was significant in groups R (101.3 ± 15.2 beats/min; *P* < 0.001) and RA (104.2 ± 12.1 beats/min; *P* = 0.003) versus group S (115.5 ± 10.7 beats/min) at 45 min after induction of anesthesia (Table 2). End-tidal sevoflurane concentration was lower in groups R (*P* < 0.001) and RA (*P* < 0.001) versus group S at 15, 30, and 45 min after induction of anesthesia (Table 3). The time to extubation was significantly prolonged in group RA (11.2 ± 2.3 min; *P* =0.004 and *P* =0.016) versus groups S (9.2 ± 2.3 min) and R (9.5 ± 2.4), but there was no significant group difference in the duration of PACU stay (*P* > 0.05) (Table 4).

**Table 2:** Mean arterial pressure and heart rate during anaesthesia

	Group S (*n* = 33)	Group R (*n* = 34)	Group RA (*n* = 35)	*P* value (overall)
MAP (mmHg)				
T0	78.9 ± 11.9	79.4 ± 12.6	80.5 ± 9.5	0.838
T15	74.0 ± 11.3	73.4 ± 8.9	74.7 ± 8.3	0.858
T30	71.5 ± 11.1	72.4 ± 10.0	73.1 ± 7.7	0.813
T45	71.8 ± 9.5	69.91 ± 9.7	71.8 ± 8.6	0.633
HR (beats/min)				
T0	112.2 ± 14.3	112.7 ± 15.4	110.2 ± 16.9	0.784
T15	118.6 ± 21.1	111.4 ± 13.9	110.8 ± 22.0	0.192
T30	115.6 ± 21.8	105.3 ± 22.5	108.5 ± 12.0	0.087
T45	115.5 ± 10.7	101.3 ± 15.2^*^	104.20 ± 12.1^*^	<0.001

Data presented as mean ± SD

Group S, patients received sevoflurane; Group R, patients received sevoflurane and continuous infusion of remifentanil; Group RA, patients received sevoflurane, continuous infusion of remifentanil, and alfentanil given 10 min before the end of surgery

*MAP* mean arterial pressure, *HR* heart rate, *T0, T15, T30, T45* time before induction of anesthesia, 15 min, 30 min, and 45 min after induction of anesthesia, respectively

^*^ *P* < 0.05 compared with group S

**Table 3:** End-tidal sevoflurane concentration

	Group S (*n* = 33)	Group R (*n* = 34)	Group RA (*n* = 35)	*P* value (overall)
Etsevo15 (Vol%)	2.5 ± 0.1	2.1 ± 0.2^*^	2.2 ± 0.2^*^	< 0.001
Etsevo30 (Vol%)	2.4 ± 0.2	2.1 ± 0.2^*^	2.1 ± 0.2^*^	< 0.001
Etsevo45 (Vol%)	2.3 ± 0.2	2.0 ± 0.2^*^	2.0 ± 0.2^*^	< 0.001

Data presented as mean ± SD

Group S, patients received sevoflurane; Group R, patients received sevoflurane and continuous infusion of remifentanil; Group RA, patients received sevoflurane, continuous infusion of remifentanil, and alfentanil given 10 min before the end of surgery

*Etsevo15, Etsevo30, Etsevo45* end-tidal sevoflurane concentration at 15 min, 30 min, and 45 min after induction of anesthesia, respectively

^*^ *P* < 0.05 compared with group S

**Table 4:** The time to extubation and PACU time

	Group S (*n* = 33)	Group R (*n* = 34)	Group RA (*n* = 35)	*P* value (overall)
Extubation time (min)	9.2 ± 2.3	9.5 ± 2.4	11.2 ± 2.3^*^	0.003
PACU time (min)	18.9 ± 5.2	18.5 ± 3.8	18.1 ± 3.5	0.770

Data presented as mean ± SD or number of patients

Group S, patients received sevoflurane; Group R, patients received sevoflurane and continuous infusion of remifentanil; Group RA, patients received sevoflurane, continuous infusion of remifentanil, and alfentanil given 10 min before the end of surgery

*PACU* postanesthesia care unit

Extubation time, time to extubation from discontinuation of sevoflurane inhalation

PACU time, duration of PACU stay

^*^ *P* < 0.05 compared with group S

The incidence of emergence agitation in group S (21/33, 64 %) was significantly higher than groups R (11/34, 32 %; *P* = 0.01) and RA (11/35, 31 %; *P* = 0.008). The numbers of patients receiving rescue alfentanil in the PACU were similar among the groups (*P* > 0.05) (Table 5).

**Table 5:** Incidence of emergence agitation and patients received rescue drug

	Group S (*n* = 33)	Group R (*n* = 34)	Group RA (*n* = 35)
Incidence of EA	21/33 (64 %)	11/34 (32 %)^*^	11/35 (31 %)^*^
Use of rescue drug	5/33 (15 %)	3/34 (8.8 %)	2/35 (6 %)

Data presented as number of patients or (%) of patients

Group S, patients received sevoflurane; Group R, patients received sevoflurane and continuous infusion of remifentanil; Group RA, patients received sevoflurane, continuous infusion of remifentanil, and alfentanil given 10 min before the end of surgery

*EA* emergence agitation, *Rescue* use of rescue alfentanil during the post-anesthesia period in patients with severe agitation (grade 4) lasting longer than 5 min

^*^ *P* < 0.05 compared with group S

## Discussion

This randomized, double-blind study investigated the effects of continuous infusion of remifentanil with or without alfentanil given 10 min before the end of surgery on the incidence of EA and recovery characteristics in pediatric patients undergoing ophthalmic surgery with sevoflurane anesthesia. The administration of continuous remifentanilreduced the incidence of EA without clinically significant hemodynamic changes or delay of recovery time. However, theaddition of alfentanil(5 μg/kg) 10 min before the end of surgery did not provide additional clinical benefit and even delayed theextubation time.

The incidence of EA largely depends on the definition, age, anesthetic technique, surgical procedure, and application of adjunct medications. Generally, it ranges from 10 to 50 %, but it is reported even up to 80 % [4, 5]. The exact mechanism of EA was not well elucidated but excitatory current activity by sevoflurane in the locus coeruleus of the central nervous system is one potential mechanism underlying the paradoxical excitatory effects of sevoflurane [23, 24]. In the practice, ophthalmic procedures have been found to be associated with higher EA as visual disturbance following surgery and eye patching would lead to higher reactivity of the child during awakening from anesthesia [25]. Therefore, suppression of EA is important issue for these patients for their safety and decreasing the needs for rescue measures.

Many studies have been conducted to findthe ways to reduce theincidence and severity of EA. One meta-analysis found that propofol, ketamine, and preoperative midazolam, fentanyl had prophylactic effects in preventing EA [26]. Comparing with these agents, remifentanil has many additional clinical benefits, including rapid onset, very short context-sensitive half-time, and lack of accumulations [16, 17, 20]. However, the efficacy of remifentanil on EA was not well evaluating to date. Some results of intraoperative use of remifentanil during adenotonsillectomy in children were promising with significantly lower incidence of EA in sevoflurane plus remifentanil group over sevoflurane, in line with current study [16, 17].

On the other hand, there were some conflicting data discouraging the use of remifentanil in this setting. The report from Choi et al. showed that remifentanil infusion led to a higher incidence of severe pain compared with N_2_O without significant reduction in EA [15]. The addition of remifentanil increased agitation accompanied by a shortened recovery time in children undergoing fiberoptic bronchoscopy under sevoflurane anesthesia [18]. However, clinical setting of their study was aside from surgery and their reports could not be directly applied to the postoperative setting. In addition, Davis et al. reported continuous infusion of remifentanilprovided faster extubation times, but associated with higher pain discomfort scores compared with bolus administration of fentanyl in pediatric patients undergoing adenotonsillectomy [20]. However, the main contention of their study is more effective intraoperative prophylactic analgesic regimens for postoperative pain control.

The addition of alfentanil in our study is based on the previous reports that the administration of highdose of remifentanil to patients during surgery is associated with a clinically small but statistically significant increase in acute pain perception after surgery [27]. And highdoseintraoperative remifentanil (0.4 μg/kg/min) could trigger postoperative hyperalgesia and anxiety compared with a lower dose (0.05 μg/kg/min) [28]. Furthermore, the report fromMendel et al., which showed alfentanil(15 μg/kg)administered during emergence from anesthesia decreased agitation without affecting the time to extubation in adult patients undergoing oral surgical procedures [21]. So, we hypothesized combination of remifentanil and alfentanil may have additive effects for reducing EA because addition of alfentail could optimize the effect of remifentanil infusion by preventing hyperalgesia. Unfortunately, alfentanil was failed to prove their efficacy on postoperative EA in our study. Different patient group, the type of surgery, and dose of alfentanil in current study would leads to this discrepancy. Our result could be interpreted as continuous remifentanil infusion itself might enough for reducing EA.

The Pediatric Anesthesia Emergence Delirium scale, proposed by Sikich et al., is a reliable and valid tool that may minimize measurement error in the clinical evaluation of EA. However, calculation of the incidence of agitation with this scale is difficult. Furthermore, ithas limited utility for the assessment of EA after ophthalmic surgery because of the presence of an item regarding eye contact, which may be interfered withbecause of an eye patch or the presence of eye ointment. Thus, we used the Watcha scale, which is a simpler toolfor use in clinical practice and may have higher overall sensitivity and specificity than other scales [29].

The current study had several limitations. First, the concentrations of sevoflurane were adjusted according to vital signs but not depth of anesthesia. However, monitoring device such as the bispectral index (BIS) was not well validated in the field of ophthalmic surgery and we believe that our approach is usual practice for many patients. Second, quantification of pain by numeric tools such as NRS was not conducted, which could not be accurately acquired from pediatric patients and might be an inherent limitation. Finally, we only tested efficacy of relative low dose of alfentanil. Further clinical research to validate higher dose of alfentanil to provide potentially other benefits for the patient should be performed in the future.

## Conclusions

In conclusion, intraoperative continuous infusion of remifentanil is effective for reducing the incidence of EA without clinically significant hemodynamic deterioration or delay of the recovery time in pediatric patients undergoing ophthalmic surgery with sevoflurane anesthesia. However, the addition of alfentanil(5 μg/kg) 10 min before the end of surgery did not provide any additional benefit and delayed the extubation time. Thus, intraoperative use of 0.1 μg/kg/min remifentanil may be effective in reducing the incidence of EA in pediatric patients undergoing ophthalmic surgery with sevoflurane anesthesia.

## Abbreviations

BIS, bispectral index; EA, emergence agitation; ET, time to extubaton; Et-sevo, end-tidal sevoflurane concentration; HR, heart rate; MAP, mean arterial pressure; PACU, post-anasthetic care unit; SD, standard deviation
